# Mobile App Use and Pregnancy Health Literacy: Cross-Sectional Study Using Bayesian Network Analysis

**DOI:** 10.2196/85403

**Published:** 2026-07-20

**Authors:** Hanne A A Spelt, Renée A Otte, Ad J M Denissen, Shihan Wang, Erin E O'Connor, Robin Neuhaus, Lucie Duracher

**Affiliations:** 1 Research & Development Philips Mother & Child Care and Women's Health Eindhoven The Netherlands; 2 Innovation & Development and AI Solutions Philips Data Science & AI Engineering Eindhoven The Netherlands; 3 Department of Information and Computing Sciences Utrecht University Utrecht The Netherlands; 4 Nested Institute for Families New York, NY United States; 5 Teaching and Learning New York University New York, NY United States

**Keywords:** health literacy, digital lifestyle interventions, mobile apps, pregnancy, women’s health, mHealth, probabilistic networks, Bayesian networks

## Abstract

**Background:**

Health literacy is crucial for pregnancy outcomes; yet, 15%-44% of pregnant women have low health literacy, negatively affecting maternal and fetal health. While pregnancy apps are increasingly used to support it, the psychological mechanisms through which they influence behavior remain unclear. Understanding these mechanisms is essential for designing effective interventions. Cognitive load theory (CLT) and the integrated model of cognitive-affective learning with media (ICALM) provide theoretical frameworks for understanding these digital interventions in pregnancy’s emotionally intense context.

**Objective:**

This study aimed to understand the psychological and social-cognitive pathways through which engagement with pregnancy apps may enhance health literacy and behaviors.

**Methods:**

This cross-sectional survey study included 998 pregnant and early postpartum women aged 18-45 years from the United States and analyzed the data using Bayesian Network (BN) modeling informed by CLT and ICALM. Participants were stratified into Pregnancy+ (Royal Philips) users (n=362), other pregnancy app users (n=507), and nonapp users (n=129). We measured digital engagement, emotions, health literacy levels, support levels and sources, and health behaviors, including physical activity, nutrition, and stress management. The BN model underwent systematic refinement through bootstrap resampling (n=1000) to evaluate arc strength, followed by k-fold cross-validation (k=5) to assess model generalizability and predictive performance.

**Results:**

In our study, pregnancy apps improve health literacy or behaviors, but not through direct pathways. Instead, they work through two indirect psychological pathways: (1) digital engagement related to positive emotions, which in turn is related to health literacy and stress management, and (2) digital engagement strengthened support networks, which in turn are related to health literacy. No direct pathways from digital engagement to health literacy or behaviors were retained. Pregnancy+ users demonstrated stronger adherence to these pathways compared with other app users and nonusers, by demonstrating more joyfulness (5.1 vs 4.7; *P*=.046), elevated support levels (4.1 vs 3.9; *P*=.03), and higher critical health literacy scores (8.4 vs 8.2 vs 7.7; *P*<.001). Pregnancy+ users also showed higher engagement frequencies (*P*<.001) and more frequently cited pregnancy apps as valuable information sources (78% vs 68%, *P*=.01) compared with users of other pregnancy apps.

**Conclusions:**

This study reveals that pregnancy apps may enhance health literacy by fostering positive emotions and strengthening support networks. This finding challenges assumptions about digital health interventions and demonstrates that apps function as psychological support systems rather than mere information sources. The findings validate CLT and ICALM within pregnancy contexts, showing that emotionally supportive apps reduce irrelevant emotional processing that consumes cognitive capacity, thereby enhancing information retention. For practice, app developers should prioritize emotional support and social connection over content volume, while health care providers should recommend apps that enhance emotional well-being. This study demonstrates BN’s value for revealing complex psychological relationships in digital health contexts.

## Introduction

### Pregnancy Health Literacy

Health literacy is a critical determinant of health outcomes [1-3], particularly for pregnant women who require adequate knowledge, motivation, and skills to access, understand, and apply health information [2-6]. Health literacy is defined as “the degree to which individuals have the capacity to obtain, process, and understand basic health information” to make health decisions [7]. In the pregnancy context, this construct operates through functional (reading or understanding information), interactive (provider communication), and critical (analytical, informed decision-making) levels [2,8].

Pregnant women have an increased need for adequate health literacy levels [3,6]. Despite the abundance of health information available during pregnancy, the prevalence of low health literacy among pregnant women remains concerning at 15%-44% [9] with direct implications for health behaviors such as physical activity [10], and prenatal care adherence [11]. This can negatively impact both maternal and fetal outcomes [12]. Low health literacy creates cascading effects throughout pregnancy care [13]—delayed prenatal care [11,14], poor comprehension of medical information [15,16], anxiety to ask questions [13] or after test results [17], and suboptimal postpartum behaviors such as reduced breastfeeding initiation [18-20]. These literacy challenges disproportionately affect racial and ethnic minorities, individuals with lower education and income, and nonnative English speakers [1,21] which contributes to maternal and neonatal health disparities [2,5].

Two psychological factors play a central role in shaping pregnancy health literacy—emotions and feelings of support. Pregnancy involves profound physiological and psychological changes [22] that can influence how women engage with health information. Evidence suggests a bidirectional relationship between emotions and health literacy. For example, pregnancy stress negatively predicts health literacy, which in turn moderates depression risk [23]. In addition, emotions may shape health behaviors during pregnancy [24-26].

Social support can mediate this relationship, which strengthens the positive effects of healthy behaviors on emotional well-being [26-28]. Also, social support may influence health literacy and behaviors [29,30], for example, when a woman feels supported by her health care provider, and better uses their health information [31].

Given the complex interplay of psychological and social factors in shaping pregnancy health literacy, digital interventions—particularly pregnancy apps—emerge as a promising solution by offering personalized, accessible support that can adapt to users’ evolving needs throughout pregnancy. While increasingly used, the psychological and cognitive mechanisms through which they influence behavior remain underexplored. Some digital interventions have shown promise in improving pregnancy health literacy [32] and outcomes [33], but the pathways through which these effects occur are not yet well-understood.

### Digital Interventions and Theoretical Frameworks for Pregnancy Health Literacy

Traditional pregnancy education interventions (in-person classes and printed materials) show inconsistent results [20,34] and face accessibility barriers [35]. Mobile apps offer flexible, on-demand health education [36,37], with 46% of prenatal apps intended for health education or self-care [38]. Evidence suggests digital interventions can improve pregnancy health literacy and foster positive attitudinal changes [32,35,39-41]. Digital engagement with specific app features—particularly educational content and self-monitoring tools—has been linked to positive outcomes [33,42]. However, emotions influence app engagement patterns, with users continuing or disengaging based on emotional support and stress levels [43-46]. Many pregnancy apps lack scientific validation, offer poor personalization, and have weak health care integration [47-49], with users often overlooking credibility and privacy risks [50-52]. Despite growing evidence of pregnancy apps’ efficacy in improving health literacy and outcomes [32,35,39-41,53], the psychological mechanisms underlying these impacts remain unclear.

The influence of digital interventions on pregnancy health literacy may be better understood through cognitive load theory (CLT), which explains how people process and retain information through working memory [54]. CLT posits that optimal learning occurs when information presentation matches available working memory capacity [54-56]. Stress and low health literacy increase mental load [57], making health behavior change more difficult [55,58].

CLT identifies 3 types of cognitive load [59]. First, intrinsic cognitive load, which reflects the effort associated with topic complexity, information volume, and previous knowledge. Second, extraneous cognitive load that reflects the effort associated with digesting information unrelated to the learning objective, for example, stemming from poor design or distractions. Third, germane cognitive load, that is, the effort associated with processing and storing the information permanently, which depends on motivation. Pregnancy involves substantial intrinsic load through complex medical information, risk assessments, and time-sensitive guidelines [4,60]. Well-designed apps can reduce extraneous load and support germane load through clean interfaces, progressive information disclosure, personalized content, and intuitive navigation—allowing users to focus cognitive resources on understanding health information rather than navigating the app itself [55,61,62]. However, the mobile environment also creates attention fragmentation through competing notifications and multitasking demands [63], risking superficial information processing that creates an “illusion of knowledge” rather than genuine health literacy development [64].

While the CLT provides a framework for the cognitive challenges of apps, pregnancy adds emotional complexity that extends beyond traditional cognitive processing. Although CLT acknowledges emotions in learning [65-68], it does not treat emotional and cognitive processing as equally important. Given that pregnant women must simultaneously process complex health information while managing uncertainty and emotional regulation, we integrate CLT with the integrated model of cognitive-affective learning with media (ICALM) [69]. ICALM assigns emotion processing equal priority to information processing, recognizing that affective and cognitive processes are intertwined and inseparable [69]. This integration is crucial because emotions affect cognitive load through multiple mechanisms [67]. They can create extraneous load through task-irrelevant emotional processing, modulate memory where positive emotions broaden and negative emotions narrow cognitive resources, influence motivation and effort allocation, and require regulation that becomes additional cognitive load. Indeed, affective neuroscience research demonstrates emotion-cognition interconnectedness at the cortical level [70,71], serving as a motivating force in learning [65,67,69,72].

This theoretical integration becomes especially relevant for pregnancy apps. Pregnancy’s emotional intensity compounds cognitive challenges [73]. Women must process health information while regulating anxiety about that information, creating dual cognitive demands, for example, learning about preeclampsia while managing fear of its complications. Learning unfamiliar concepts while managing anxiety can overwhelm processing capacity and reduce motivation for meaningful learning [73,74]. Research suggests that well-designed antenatal education can effectively manage these demands, improving health literacy while reducing fears [75]. Together, CLT and ICALM provide a comprehensive framework for understanding how pregnancy apps can optimize both cognitive processing and emotional engagement.

### Using Bayesian Networks to Uncover Psychological Mechanisms

Pregnancy health literacy encompasses a complex interplay of knowledge, skills, and contextual factors that enable women to navigate prenatal health information and make informed decisions. Probabilistic networks, such as Bayesian networks (BNs), have emerged as powerful tools for handling uncertainty in complex domains [76,77], capable of uncovering causal psychological relationships beyond simple correlations [78] and mapping psychological constructs as complex network structures [76]. We selected BNs over traditional methods, such as structural equation modeling, because our research aims to explore pathways through data rather than confirm prespecified relationships. Furthermore, BNs allow researchers to include evidence-based information in the discovery process [77-79].

BNs have proven valuable across diverse psychological domains, including personality [80], health-related quality of life, general intelligence [81], attitudes [82], and mindfulness [83]. In pregnancy care specifically, they show practical promise through integrated mobile monitoring systems [84]. The novelty of our approach lies in systematically combining psychological theories, such as CLT and ICALM, with survey data within the network structure, offering an ideal methodology to uncover the psychological and social-cognitive pathways through which mobile app engagement may enhance health literacy during pregnancy.

### Study Aims

This study investigates how digital engagement with pregnancy apps contributes to health literacy development through psychological and social-cognitive mechanisms. Using cross-sectional survey data from 1066 US pregnant and early postpartum women in the United States, this study aims to (1) compare health literacy levels between Pregnancy+ app (Royal Philips) users, other app users, and nonusers; (2) identify psychological and social-cognitive pathways through which digital engagement influences health literacy and health behaviors using theory-informed BN modeling; and (3) validate these pathways to inform the design of more effective digital health literacy interventions. By exploring the mechanisms through which cognitive load and emotions influence health literacy, we aim to provide actionable insights for developing pregnancy apps that effectively support health literacy development.

## Methods

### Study Design

This study used a cross-sectional observational survey design to investigate psychological and social-cognitive pathways through which pregnancy app engagement influences maternal health literacy. Survey data from 1066 pregnant and postpartum women were analyzed using BN methodology, which integrates theoretical frameworks (CLT and ICALM) with data-driven discovery to model complex psychological relationships. This approach follows established best practices in BN construction for psychological research [76,78]. This study adheres to the STROBE (Strengthening the Reporting of Observational Studies in Epidemiology) guidelines for cross-sectional studies [85]. A detailed checklist demonstrating compliance with the guidelines is provided in Multimedia Appendix 1.

### Participants

This study targeted females aged 18-45 years who were either currently pregnant or mothers of children aged 0-12 months in the United States. A carefully constructed stratified sampling approach ensured comprehensive representation across different user groups and demographic characteristics. A total sample of 900 respondents was planned, with stratified sampling to ensure representation across three user groups: (1) Pregnancy+ app users (n=300), (2) users of other pregnancy apps (n=300), and (3) nonusers of any pregnancy apps (n=300). No formal power analysis was conducted, as BN modeling is exploratory rather than confirmatory, and established power analysis frameworks for BN structure learning are limited. The target of 300 participants per group was determined to ensure sufficient cell sizes for stable conditional probability estimation within the discretized network nodes, while allowing balanced stratification. To qualify as app users for analytical purposes, participants needed to report using their respective pregnancy app for more than 2 months, with a usage frequency of at least several times per week. Within each user group, equal distribution across 3 ethnic categories was implemented—White (n=100), Hispanic (n=100), and Black (n=100). Due to limited panel availability, Hispanic participants were recruited on a best-effort basis to approximate target quotas. Additional quota sampling ensured balanced representation between pregnant participants (n=500) and postpartum mothers (n=400). Soft quotas were also established to monitor representation across health insurance types (commercial or private and Medicaid), pregnancy stages (trimesters), and age distribution to approximate national representation within each ethnic group. No poststratification weighting was applied; the quota sampling approach was designed to ensure balanced representation at the recruitment stage rather than through post hoc statistical adjustment.

### Ethical Considerations

This study did not require approval from a Medical Research Ethics Committee under the Dutch Medical Research Involving Human Subjects Act [86], as it involved only anonymous online survey research with adult participants. All participants provided informed consent before participation (refer to Procedure section for details). Participants were compensated for their time through research panel credits redeemable for gift vouchers, equivalent to approximately €0.50-€2.40 (€1=US $1.17 approximately at the time of study administration) per participant, depending on panel membership.

### Procedure

The survey was administered online through a professional market research agency on behalf of Royal Philips between October and December 2024. Participants were recruited from established research panels and provided with informed consent detailing the study’s purpose, confidentiality provisions, and their rights as participants (eg, right to withdraw and data confidentiality). After confirming consent, participants completed the screening questions to determine eligibility based on the established quotas.

Eligible participants proceeded through the survey modules in a fixed sequence. Quality control measures were implemented throughout the survey, including attention checks and open-ended validation questions to ensure data integrity. The survey platform ensured appropriate question routing based on participant characteristics (pregnant vs postpartum) and app usage status. After data collection, the research agency provided us with the deidentified participant data. Of 1066 complete survey responses received from the research agency, 68 participants who selected “prefer not to say” for one or more health behavior variables were excluded, resulting in a final analytical sample of 998 participants. Because the research agency delivered only fully completed surveys, there were no missing data within the analyzed variables; a missing completely at random test and multiple imputations were therefore not applicable.

### Intervention

Pregnancy apps used by participants were generally free to download with optional premium features. They typically provided features such as educational content per trimester, weight trackers, contraction timers, and tools for sharing updates with partners or family members. Pregnancy+ additionally offered a visually structured interface featuring week‑by‑week information on fetal development, including interactive 3D models. These design elements present information in a more visual format compared with the predominantly text‑based or static‑image layouts seen in many other pregnancy apps, which may contribute to different engagement patterns.

### Measures

#### Overview

The demographic information encompassed participants’ age, ethnicity, state of residence, household composition, socioeconomic indicators, and gestational age. Parity was assessed as a binary variable, with participants classified as nulliparous (first pregnancy) or primiparous (second pregnancy or beyond). Overall, the survey contained a maximum of 63 questions. Not all questions were presented to every participant; routing logic ensured relevance based on previous responses. Except for the health literacy items, all measures were purpose-designed for this survey and have not been independently validated. The complete questionnaire is provided in Multimedia Appendix 2.

#### Digital Engagement

Digital engagement information included pregnancy apps downloaded and, if applicable, precise timing of downloads (before pregnancy, in the first trimester, second trimester, or third trimester), frequency of use (ranging from several times a day to less often than 1-2 times every 3 months), and duration of engagement (ranging from less than 1 month to more than 9 months). A composite variable was created based on usage frequency and duration, categorizing participants into no, low, medium, or high engagement groups.

#### Emotions

Emotions across pregnancy trimesters were measured separately using semantic differential sliders on 7-point Likert scales measuring 6 bipolar emotional dimensions purpose-designed for this study. These dimensions ranged from negative to positive and included stressed-excited, worried-hopeful, angry-calm, distressed-joyful, sad-at ease, and lonely-connected. Participants rated their emotional experiences during each trimester, and if applicable, also during postpartum.

#### Health Literacy

Health literacy was measured using 6 items developed for this study based on Nutbeam’s 3-level framework [2,3], rated on 10-point Likert scales assessing functional health, interactive health literacy, and critical health literacy.

#### Support Level

Support level was measured using a single study-specific 5-point Likert scale where participants indicated their agreement with feeling supported during pregnancy (retrospectively for postpartum participants), ranging from strongly disagree to strongly agree.

#### Support Sources

Support sources were assessed using a study-specific multiple-choice question identifying 13 sources that provided informational support during pregnancy, including partner, family and friends, health care providers, pregnancy apps, websites or blogs, social media or influencers, documentaries or videos, books or magazines, local groups, health insurance resources, and other sources.

#### Health Behaviors

Health behaviors were assessed using study-specific items covering physical activity (150 min of moderate exercise weekly), dietary practices (balanced nutrition intake), and stress management techniques (meditation and breathing exercises). Participants indicated their behavioral adoption status across 4 categories—adopted before pregnancy, adopted during pregnancy, trying to adopt, or not trying to adopt.

### Statistical Analysis

#### Overview

Data were analyzed using R Studio (version 4.4.2; Posit PBC), with a significance level set at α=.05 and using packages *bnlearn* for BN construction and validation [87,88], *Rgraphviz* for network visualization [89], and *ggplot2* [90], *dplyr* [91], *carData* [92], and effects [93] for data manipulation and supplementary analyses. Group comparisons across app usage groups were performed using chi-square tests for categorical variables and independent Welch *t* tests for continuous variables.

#### BN Approach

Survey data from 998 pregnant and postpartum women were analyzed using BN methodology, which integrates theoretical frameworks (CLT and ICALM) with data-driven discovery to model complex psychological relationships. This approach follows established best practices in BN construction for psychological research [76,78]. BNs consist of directed acyclic graphs, where nodes represent variables and directed arcs represent relationships among them [79], which can be expressed in quantitative probabilistic terms [77]. The network represents joint probability distributions in a compact way, providing a flexible representation of variable dependencies through network structure [94]. A definition of BNs, including their mathematical expressions, can be found in the study by Lee and Abbott [77]. In this work, we use BNs to uncover effective variables of psychological mechanisms and construct their relations from survey data.

We integrated theoretical frameworks with data-driven discovery to address unmeasured confounders, which is a common problem in psychology where social, environmental, and economic factors affect results [95]. This integration of evidence-based information into BN construction is standard and explicitly recommended practice [79]. By incorporating theoretical constraints from CLT, ICALM, and pregnancy literature alongside our survey data, we modeled complex relationships between digital engagement, emotions, support systems, and health outcomes. This approach guides the network structure based on established theory while allowing data-driven discovery of unexpected relationships, reducing computational demands by focusing on theoretically meaningful connections [77].

In BNs, confounding is addressed through the network structure itself—a variable that influences both an exposure and an outcome appears as a parent node of both, and its effect is accounted for through the conditional probability tables. In our model, parity was positioned as an upstream variable influencing both digital engagement and emotions, thereby serving a role analogous to a confounder in traditional analyses. Demographic variables (age, ethnicity, and socioeconomic status) were addressed through the stratified sampling design rather than included as network nodes, to maintain model parsimony while ensuring balanced group representation.

#### Network Construction and Validation

A BN was constructed and validated through 3 phases [79].

#### Node Specification and Preprocessing

Variables that should be included in the BN as *nodes* were selected by connecting survey data outcomes with key factors from the CLT, ICALM, and pregnancy literature. Principal component analysis (PCA) was applied for dimensionality reduction where needed, with data suitability assessed using the Kaiser-Meyer-Olkin measure and Bartlett test of sphericity [96,97]. Components were retained based on eigenvalues >1 (Kaiser criterion), and factor loadings were examined to confirm unidimensionality. All continuous variables were discretized into categorical variables distribution histograms with approximately equal group sizes to enable flexible modeling of nonlinear relationships and threshold effects [98].

#### Theory-Informed Network Structure Formulation

The initial network structure was specified based on theoretical relationships from CLT, ICALM, and existing pregnancy literature, establishing theoretical relationships between risk factors, interventions, psychological manifestations, and health behavior outcomes. A constraint-based approach with the Peter-Clark algorithm implemented in *bnlearn* was used to further construct the BN from the survey data [88]. Theoretical constraints were incorporated through whitelist constraints to ensure theoretically meaningful connections while allowing data-driven discovery of additional relationships (Multimedia Appendix 3). This integration of theory and data represents the strength of BN methodology, while maintaining theoretical validity, while remaining open to unexpected empirical relationships.

#### Network Validation

First, the initial network structure underwent systematic empirical refinement to optimize the balance between theoretical validity and statistical support. This iterative process involved (1) bootstrap resampling (n=1000) to evaluate arc strength using chi-square statistics and Bayesian information criterion (BIC) scores; (2) sequential removal of arcs with weakest empirical support, beginning with the lowest-scoring connections; (3) refitting and validation after each removal using k-fold cross-validation (k=5) to assess impact on log-likelihood loss; and (4) preservation of theoretically essential relationships through whitelist constraints throughout the refinement process. The refinement continued until further arc removal either violated theoretical constraints or resulted in deteriorating model performance.

Second, network validation was performed through k-fold cross-validation (k=5) to assess model generalizability and obtain unbiased estimates of predictive performance. Model performance was evaluated using log-likelihood assessment, which measures how well the probabilistic model predicts observed outcomes [88], with lower values indicating better model fit and predictive accuracy. The final optimized network was compared against the initial theory-informed structure and alternative configurations to demonstrate improvement in model performance.

The iterative nature of BN construction means that structural decisions are both methodological choices and findings. We report on the construction process in Results to maintain transparency about data-driven refinements.

## Results

### Participant Descriptives

A total of 998 participants were included in the analysis (Table 1). The sample comprised women aged 18 to 45 years (mean 29.8, SD 7.1 y), with the majority identifying as White (n=424, 42%), followed by Black (n=305, 31%), Hispanic (n=177, 18%), and other (n=92, 9%). The sample included 450 (45%) postpartum mothers and 548 (55%) currently pregnant women (n_T1_=106, n_T2_=267, and n_T3_=175). Nulliparous mothers comprised 334 (33%) participants of the total sample, whereas 664 (67%) were primiparous. Among the participants reporting using pregnancy apps (n=869, 87%), the most frequently used apps included Pregnancy+ (n=362), BabyCenter (Ziff Davis; n=174), and What to Expect (Ziff Davis; n=140). For comparative analysis, participants were categorized into following three groups based on their app usage patterns: (1) participants who used Pregnancy+ as their primary app, (2) participants who used other pregnancy apps, and (3) participants who either never downloaded a pregnancy app or did not use one regularly (less than several times per week) or for an extended period (less than 1 month).

**Table 1:** Key outcomes for the total sample and per app usage group.

Item		Total (N=998)	Pregnancy+ users (n=362)	Other app users (n=507)	Non-app users (n=129)	*P* value^a^ Pregnancy+ vs other	*P* value^a^ Pregnancy+ vs none
**Frequency of app use, n (%)**						<.001	—^b^
	Several times a day	306 (31)	142 (39)	164 (32)	—		
	Several times a week	395 (40)	187 (52)	208 (41)	—		
	Once a week	112 (11)	26 (7)	86 (17)	—		
	1-2 times per month	34 (3)	6 (2)	28 (6)	—		
	1-2 times every 3 months	12 (1)	0 (0)	12 (2)	—		
	Less often	10 (1)	1 (0)	9 (2)	—		
Months of app use, mean (SD)		6.9 (3.7)	6.4 (3.4)	7.3 (3.9)	—	<.001	—
**Emotions, mean (SD)**							
	Excited	4.6 (1.6)	4.7 (1.6)	4.5 (1.6)	4.4 (1.6)	.14	.12
	Hopeful	4.5 (1.6)	4.6 (1.6)	4.5 (1.6)	4.4 (1.6)	.41	.23
	Calm	5.1 (1.4)	5.1 (1.4)	5.0 (1.4)	4.9 (1.5)	.97	.10
	Joyful	5.0 (1.5)	5.1 (1.4)	5.0 (1.5)	4.7 (1.5)	.68	.046
	At ease	4.8 (1.5)	4.8 (1.4)	4.7 (1.5)	4.6 (1.6)	.77	.21
	Connected	5.0 (1.6)	5.0 (1.6)	4.9 (1.6)	4.9 (1.7)	.70	.53
**Health literacy, mean (SD)**							
	Functional	7.8 (2.0)	7.9 (2.0)	7.7 (2.0)	7.9 (2.0)	.17	.99
	Interactive	8.5 (2.0)	8.6 (1.8)	8.4 (2.1)	8.6 (1.7)	.06	.77
	Critical	8.2 (1.6)	8.4 (1.5)	8.2 (1.7)	7.7 (1.8)	.27	<.001
Support level, mean (SD)		4.0 (1.1)	4.1 (1.0)	3.9 (1.1)	3.9 (1.1)	.03	.06
**Support sources, n (%)**							
	Partner	421 (42)	153 (42)	210 (41)	58 (45)	.86	.67
	Family and friends	611 (61)	223 (62)	302 (60)	86 (67)	.59	.36
	Health care providers	542 (54)	199 (55)	274 (54)	69 (53)	.84	.85
	Pregnancy apps	627 (63)	282 (78)	345 (68)	—	.01	—
	Pregnancy websites	448 (45)	158 (44)	230 (45)	60 (47)	.11	.43
	Social media	415 (42)	164 (45)	213 (42)	38 (29)	.37	.002
	Documentaries, videos	245 (25)	99 (27)	120 (24)	26 (20)	.25	.14
	Pregnancy books	323 (32)	115 (32)	170 (34)	38 (29)	.64	.71
	Local groups	110 (11)	37 (10)	65 (13)	8 (6)	.29	.24
	Health insurance company	129 (13)	49 (14)	68 (13)	12 (9)	≥.99	.27
	Health insurance resources	350 (35)	126 (35)	190 (37)	34 (26)	.46	.10
	Other, please specify	9 (1)	3 (1)	6 (1)	0 (0)	.87	.71
**Physical activity, n (%)**						.14	.20
	Adopted before pregnancy	299 (30)	95 (26)	162 (32)	42 (33)		
	Adopted during pregnancy	271 (27)	102 (28)	134 (26)	35 (27)		
	Trying to adopt	354 (35)	143 (40)	171 (34)	40 (31)		
	Not trying to adopt	74 (7)	22 (6)	40 (8)	12 (9)		
**Nutrition, n (%)**						.70	.13
	Adopted before pregnancy	300 (30)	106 (29)	156 (31)	38 (29)		
	Adopted during pregnancy	304 (30)	115 (32)	145 (29)	44 (34)		
	Trying to adopt	328 (33)	122 (34)	173 (34)	33 (26)		
	Not trying to adopt	65 (7)	19 (5)	33 (7)	13 (10)		
**Stress management, n (%)**						.18	.09
	Adopted before pregnancy	229 (23)	81 (22)	115 (23)	33 (26)		
	Adopted during pregnancy	280 (28)	118 (33)	132 (26)	30 (23)		
	Trying to adopt	425 (43)	144 (40)	228 (45)	53 (41)		
	Not trying to adopt	64 (6)	19 (5)	32 (6)	13 (10)		

^a^*P* values represent comparisons groups using chi-square tests for categorical variables and independent Welsch *t* tests for continuous variables, P+ = Pregnancy+ users, other = other app users, none = nonapp users.

^b^Not applicable.

### Network Node Characteristics

The network comprised 9 nodes—parity, emotions, support level, support sources, digital engagement, health literacy, stress management, physical activity, and nutrition (Tables 1 and 2). Moreover, 4 variables required preprocessing through PCA to create unidimensional constructs suitable for network modeling; the emotions PCA revealed 1 primary component explaining 41% of variance with factor loadings ranging from 0.366 to 0.462, confirming a unidimensional emotional valence construct. The support sources PCA revealed 1 primary component explaining 77% of the cumulative variance, with all items demonstrating factor loadings ranging from 0.22 to 0.42, representing overall support usage. The health literacy PCA revealed 1 primary component explaining 55% of the variance, confirming a unidimensional construct representing overall health literacy with factor loadings ranging from 0.237 to 0.483. The continuous variables were discretized in categories based on their distribution (Multimedia Appendix 4).

**Table 2:** Nodes requiring preprocessing with their values as used in the Bayesian network model.

Nodes^a^		Range	n (%)
**Emotions**			
	Negative	1.00 to 3.73	200 (20)
	Slightly negative	3.73 to 4.44	201 (20)
	Neutral	4.44 to 5.17	201 (20)
	Slightly positive	5.17 to 5.94	196 (20)
	Positive	5.94 to 7.00	200 (20)
**Support sources**			
	Very low	–1.43 to –0.64	206 (21)
	Low	–0.64 to –0.22	193 (19)
	Moderate	–0.22 to 0.16	200 (20)
	High	0.16 to 0.63	199 (20)
	Very high	0.63 to 1.66	200 (20)
**Digital engagement**			
	No	–1.00 to 0.00	129 (13)
	Low	0.00 to 11.00	173 (17)
	Medium	11.00 to 17.00	400 (40)
	High	17.00 to 24.00	296 (30)
**Health literacy**			
	Low	2.33 to 7.17	216 (22)
	Below average	7.17 to 8.17	214 (21)
	Average	8.17 to 8.83	200 (20)
	Above average	8.83 to 9.50	206 (21)
	High	9.50 to 10.0	162 (16)

^a^Nodes not requiring preprocessing are parity, support level, physical activity, nutrition, and stress management, which are reported in the text and Table 1.

### Network Structure Results

**Figure 1:**
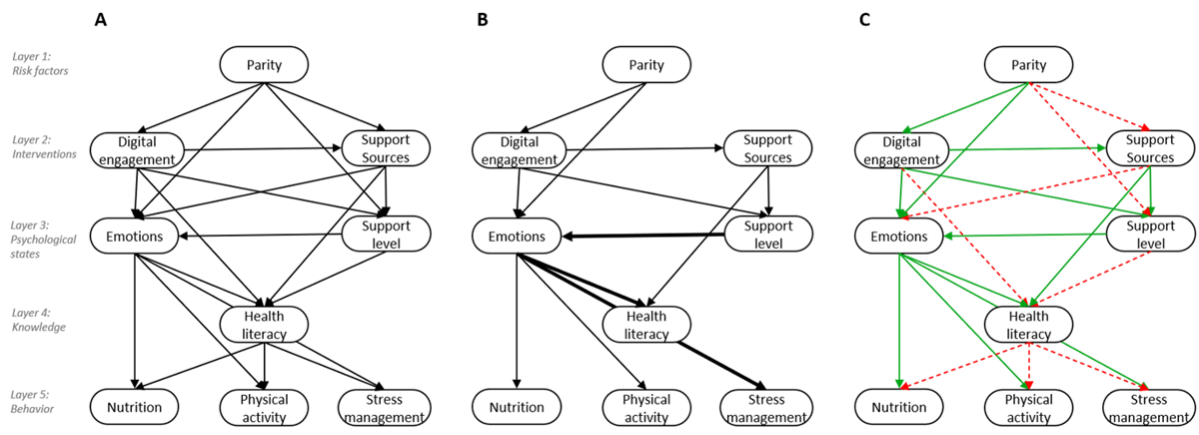
Bayesian network representations showing theory-informed structure and empirical validation. (A) Initial theory-informed network based on cognitive load theory, integrated model of cognitive-affective learning with media, and evidence-based information with a hierarchical layer structure. (B) Final empirical network after data-driven refinement (n=998), with line thickness indicating relationship strength. (C) Comparison highlighting removed arcs (red dashed) and preserved arcs (green solid).

The initial theoretical network established a hierarchical structure with risk factors influencing interventions and psychological manifestations, which subsequently affected health behavior outcomes (Figure 1A). The empirical network derived from data-driven refinement maintained this theoretical foundation while optimizing statistical significance and model parsimony (Figure 1B).

Several weak connections present in the initial theoretical model were eliminated during refinement, including direct pathways from parity to outcome variables. The final refined network retained key pathways that demonstrated both statistical significance and theoretical importance. The final refined network retained key pathways that demonstrated both statistical significance and theoretical importance (Table 3).

The strongest relationship identified was between emotions and stress management (*P*<.001, BIC=−5.6). Similarly, emotions showed a strong direct relationship with health literacy (*P*<.001, BIC=176.9). Support mechanisms emerged as interconnected constructs, with support level influencing both support source diversity (*P*<.001, BIC=160.9) and emotions (*P*<.001, BIC=297.0). Digital engagement influenced outcomes through 2 primary indirect pathways—affecting support source usage (*P*<.001, BIC=147.9), which subsequently impacted health literacy (*P*<.001, BIC=205.6), and directly influencing emotions (*P*=.01, BIC=337.7), which then affected multiple health behaviors, including physical activity and nutrition. No direct pathways from digital engagement to health behaviors were retained in the final model. Full arc strength statistics for all retained arcs are presented in Table 3.

**Table 3:** Arc strengths in the final refined network model.

From	To	*P* value (*χ*^2^ test)^a^	Strength (BIC^b^)
Emotions	Stress management	<.001	−5.6
Emotions	Health literacy	<.001	176.9
Emotions	Physical activity	<.001	21.9
Emotions	Nutrition	<.001	21.5
Support level	Support sources	<.001	160.9
Support level	Emotions	<.001	297.0
Support sources	Health literacy	<.001	205.6
Digital engagement	Support sources	<.001	147.9
Digital engagement	Support level	.09	31.6
Digital engagement	Emotions	.01	337.7
Parity	Emotions	.08	220.4
Parity	Digital engagement	.21	8.0

^a^*P* values derived from Pearson chi-square tests on arc strength, as computed by the arc.strength() function in bnlearn with criterion=“ *χ*^2^.”

^b^BIC: Bayesian information criterion.

### Network Validation Results

Model validation demonstrated substantial improvement following network refinement. The initial theory-driven network demonstrated an expected log-likelihood loss of 15.51, indicating poor model performance and substantial model uncertainty. Following systematic refinement based on arc strength analysis using the chi-square statistics and BIC values, the iterative removal of statistically unsupported arcs resulted in progressive improvement in model performance. The final model with 7 arcs pruned (Figure 1B) achieved an improved expected log-likelihood loss of 11.93, representing a meaningful improvement in model fit and predictive accuracy. Pruning more arcs resulted in a higher log-likelihood loss. This 23% reduction in log-likelihood loss means the refined model substantially outperforms the initial theory-only structure in predicting the observed data, while retaining the theoretically motivated core relationships.

## Discussion

### Principal Findings

This study reveals a critical finding about how digital interventions influence pregnancy health literacy; digital engagement with pregnancy apps does not directly improve health literacy or health behaviors. Instead, apps, including Pregnancy+, work through indirect psychological pathways by enhancing emotional well-being and creating feelings of support, which then lead to improved health literacy and behaviors. This finding fundamentally challenges the assumption that providing information directly causes behavior change. Using BN analysis of survey data from 998 pregnant and postpartum women, we identified 2 pathways linking digital engagement to health literacy. First, digital engagement was associated with more positive emotional states, which in turn related to higher health literacy and stress management behaviors; and second, digital engagement was associated with broader support source usage, which subsequently linked to health literacy. These findings validate CLT and ICALM within pregnancy contexts and demonstrate how pregnancy apps function as psychological support systems rather than mere information delivery platforms.

Emotions emerged as the primary mediator between digital engagement and health outcomes, with the strongest relationships linking emotions to both stress management and health literacy. This aligns with emerging evidence linking mental health to pregnancy health literacy [99] and health behaviors [24-26]. The pathway from digital engagement to emotions to health literacy suggests that emotionally supportive apps reduce task-irrelevant emotional processing that consumes cognitive capacity, thereby enhancing information retention. Supporting this interpretation, Pregnancy+ users reported greater feelings of joy compared with nonapp users and more frequently cited pregnancy apps as useful information sources (282/362, 78% vs 345/507, 68% for other app users). Although our cross-sectional design precludes causal claims, these patterns suggest a dose-response relationship where more engaged users demonstrate better emotional outcomes and more active information-seeking behaviors. The second critical pathway evolved around support. Digital engagement influenced how many support sources women used, which then impacted their health literacy. Additionally, feeling supported influenced both support source variety and emotions. The most frequently cited support sources all facilitate interpersonal connection: pregnancy apps (627/998, 63%), family and friends (611/998, 61%), and health care providers (542/998, 54%). These findings complement research showing that social support mediates the relationship between mental health and healthy behaviors during pregnancy [27,28]. This interconnected support-emotion-literacy network suggests that effective pregnancy apps function as social connectors that enhance broader support networks rather than isolated information sources. Notably, Pregnancy+ users demonstrated stronger alignment with both identified pathways compared to other app users and nonusers, evidenced by higher joyfulness scores, higher support levels, and higher critical health literacy scores. These group differences suggest that app design features may modulate the strength of the emotion and support pathways.

### Theoretical Implications

Our findings provide empirical validation of CLT and ICALM within the pregnancy context, confirming that emotional processing operates as an independent and equally weighted pathway alongside cognitive processing in shaping health literacy outcomes, and that digital engagement influences these pathways indirectly rather than directly. The central role of emotions in our network supports CLT advances on emotion-cognition interconnectedness [70,71], emotions as a motivating force in learning [65,67,72], and mechanisms through which emotions impact cognitive load [67]. The strong direct effect of emotions on health literacy and stress management suggests that negative emotions create unnecessary extraneous cognitive load, when, for example, anxiety about fetal health competes with information processing, while positive emotions facilitate learning. The pathway from digital engagement to emotions to health literacy suggests that emotionally supportive pregnancy apps may reduce irrelevant emotional processing that typically uses up cognitive capacity and enhance information retention.

Notably, digital engagement did not demonstrate direct strong effects on health literacy or health behaviors. This challenges common beliefs about how digital health interventions work, such as “providing good information directly leads to behavior change,” “more engagement leads to better health outcomes” [42], or “learning educational content is what drives improvements in health literacy and behaviors” [100]. The absence of direct pathways from digital engagement to health literacy and behaviors aligns with ICALM’s core principle that emotional processing operates with equal priority to cognitive processing [67]. The interconnected support-emotion-learning pathways exemplify ICALM’s principle that cognitive and affective processes are inseparable rather than competing systems. This integrated processing appears especially pronounced during pregnancy, where hormonal changes, physical discomfort, and life transitions create emotional intensity that distinguishes pregnant women from other populations studied in CLT and ICALM research.

The finding that parity influences pregnancy outcomes through emotions and digital engagement aligns with CLT’s predictions about novice versus expert learners. First-time mothers lack existing pregnancy schemas, requiring explicit instruction to prevent extraneous cognitive load [74]. This drives higher digital engagement as they build foundational knowledge while simultaneously creating stronger emotional responses due to uncertainty. The network shows that these emotions subsequently influence health literacy and behaviors, suggesting digital interventions may be particularly valuable for nulliparous women who benefit most from cognitive scaffolding and emotional support.

### Implications for Pregnancy App Design

Our findings necessitate fundamental shifts from information-focused to emotion-aware pregnancy app design that addresses both cognitive and emotional pathways to health literacy. Rather than prioritizing content volume or educational comprehensiveness, apps should create emotionally supportive experiences that facilitate natural behavior change processes. This approach recognizes that women use pregnancy apps for different purposes—cognitive information-seeking (particularly among postpartum women and those with pregnancy risks [101]) and emotional regulation strategies, such as seeking uplifting content when distressed [102]. Research suggests digital interventions serve emotional reassurance purposes rather than clinical data; for example, women predominantly use apps to track baby movements and weight rather than blood pressure or glucose [101]. This means moving away from the assumption that “more information equals better outcomes” toward understanding that emotional well-being enables women to effectively process and apply health information.

Apps should personalize content based on cognitive load and emotions. Since cognitive load demands are context-specific, pregnancy apps should adapt complexity based on user state. During high-stress periods, apps should minimize cognitive demands while maintaining supportive presence, while during calm periods, apps can present more complex information and encourage deeper processing. This adaptive approach represents a significant advancement over static information delivery models currently dominating digital health interventions. Implementation may become feasible through just-in-time adaptive interventions delivering personalized, emotionally appropriate content dynamically [101], combined with psychophysiological integration and emotion detection via wearables enabling real-time adaptation [103,104].

While emotion-aware systems offer more pleasant, efficient user experiences, they raise ethical concerns [105]. Autonomy concerns arise because unobtrusive measures prevent users from controlling emotional information feeding into the system. Trust issues emerge because while users expect apps to support their well-being, systems may oversimplify the complex, context-dependent nature of emotional episodes [106]. These concerns are particularly acute for pregnant populations, given their altered emotions due to hormonal changes and increased vulnerability.

Digital engagement measured through traditional metrics (session duration, page views, and clicks) may be suboptimal effectiveness indicators. The relationship between digital engagement and emotions is more complex than simple usage metrics suggest. Smartphone usage patterns predict emotions with 86.17% accuracy for depression, anxiety, and stress [44], while emotions drive app selection behaviors (sadness prompting social media use and joy leading to content sharing) [102]. This suggests users actively use apps as emotion regulation strategies, creating opportunities for timely, emotionally appropriate interventions during vulnerable periods like pregnancy. The BN offers a more nuanced understanding of how digital tools enhance feelings of support and emotional well-being, revealing interconnected pathways that explain why simple usage metrics often fail to predict behavioral changes. Meaningful engagement should be measured by emotional support facilitation, support network enhancement, and emotional improvements rather than usage quantity alone.

The finding that digital engagement relates to outcomes through support suggests apps can actively facilitate connections to health care providers, family, and peers while enhancing emotional connection quality. This ecosystem approach may optimize cognitive processing by connecting new information to users’ existing support structures. These findings have important clinical implications—pregnancy apps can positively impact health literacy and behaviors, promoting hybrid care models combining traditional care with digital interventions. Providers may recommend high-quality pregnancy apps that contribute to patients’ emotional well-being and support usage, while, from a population health perspective, promoting apps like Pregnancy+ could improve outcomes through enhanced support networks.

### Methodological Contribution

This study demonstrates the value of BNs for revealing complex psychological ecosystems in pregnancy. Networks are particularly valuable in psychology for identifying complex factor-behavior relationships, providing holistic approaches by validating research questions and revealing how changes cascade through outcomes [77,78]. They offer advantages over traditional methods by allowing integration between evidence-based knowledge and intuitive graphical structures that make models easier to understand [77-79]. Integrating domain knowledge with observational survey data from 998 pregnant and postpartum women represents a significant methodological advancement, revealing relationships that traditional studies might miss. The validated network reflects participants’ self-reported experiences in naturalistic, nonclinical settings, providing practical relevance for immediate app development and clinical practice implementation.

### Limitations

Several limitations should be considered when interpreting this study’s findings. To start, our emotional assessment captured general valence and arousal across pregnancy trimesters rather than app-specific emotional responses or discrete emotional episodes. Researchers distinguish between emotional episodes (contextually constructed and dynamic responses) and mood (ongoing and free-floating affect) [107]. Although these constructs influence each other, emotional episodes are more likely to impact cognitive load due to their dynamic nature and higher psychological complexity [107] operating on the same timescale as cognitive load [69]. Additionally, an important aspect of emotional life may be the simultaneous experience of multiple emotions within a given time and context [69], whereas our study combined multiple retrospective ratings per trimester into a single valence measure for the BN. Future research should differentiate between in-the-moment emotional episodes during app usage and broader mood states, incorporating validated questionnaires (eg, 6 basic emotions [108] or the positive and negative affect scale [109]) to better understand how pregnancy apps influence various emotional aspects.

Our specific implementation of BNs may have introduced constraints. We developed a state model rather than a temporal one, limiting our ability to capture dynamic changes over time. Temporal modeling would better represent real-world situations where interactions between digital engagement, emotions, and health literacy are fluid and evolving. The model may exhibit causal independence issues, as multiple parent nodes (digital engagement, parity, and support level) influence emotions through potentially interactive rather than independent processes. The model might have performed better with additional psychological determinants such as self-efficacy, motivation, attitudes, and coping skills. These omissions may limit understanding of complete causal pathways.

The cross-sectional design prevents causal inferences despite BNs’ ability to represent causal relationships. Longitudinal designs with repeated measures would strengthen causal claims and better capture the dynamic nature of emotion-cognition-behavior relationships throughout pregnancy. Also, data collection relied primarily on retrospective, nonvalidated questionnaires. More ecologically valid data could be obtained through real-time observational designs. App usage analytics examining engagement with specific features, incorporating different emotional design elements, would provide more nuanced digital engagement measures than self-reported frequency and duration. Similarly, physiological measures during actual app usage could capture cognitive load and emotional episodes as they occur rather than relying on retrospective trimester ratings. Such approaches would enable BNs to reveal more precise mechanisms.

### Conclusion

This study demonstrates that pregnancy apps influence health literacy and behaviors through indirect psychological pathways—enhancing emotional well-being and strengthening support networks—rather than through direct information delivery. This finding fundamentally challenges traditional digital health intervention models and necessitates shifts toward emotion-aware, psychologically informed design approaches.

For app developers, the findings indicate that emotional support and social connection should be prioritized over content volume. Pregnancy apps can function as psychological support systems by reducing cognitive load through emotional regulation. For health care providers, results suggest they could recommend apps that enhance emotional well-being and facilitate support networks. For researchers, the successful application of BNs demonstrates their value for uncovering complex psychological mechanisms in digital health contexts.

Future research should use longitudinal designs with real-time emotional measures during app usage, investigate how specific app features influence the emotion-support-literacy pathways, and examine these mechanisms across diverse populations and health contexts. This work opens new avenues for developing emotionally responsive digital health technologies that could transform maternal health care and extend to other vulnerable populations by navigating complex health decisions.

## Appendix

Multimedia Appendix 1 STROBE Checklist for Cross-Sectional Studies.

Multimedia Appendix 2 Survey questions.

Multimedia Appendix 3 Whitelist constraints for Bayesian network arc specification.

Multimedia Appendix 4 Histograms with discretization of emotions, support sources, digital engagement and health literacy.

## Abbreviations

BIC: Bayesian information criterion
BN: Bayesian network
CLT: cognitive load theory
ICALM: integrated model of cognitive-affective learning with media
PCA: principal component analysis
STROBE: Strengthening the Reporting of Observational Studies in Epidemiology

## Data Availability

Due to the sensitive nature of the survey questions, participants were assured that their raw data would remain confidential and would not be shared publicly. Accordingly, the raw study data are not publicly available to remain compliant with the consent provided by respondents. Researchers interested in further information about the data may contact the corresponding author.
