# Supplementary material for: Mobile App Use and Pregnancy Health Literacy: Cross-Sectional Study Using Bayesian Network Analysis
Source: J Med Internet Res. 2026 Jul 20;28:e85403. doi: 10.2196/85403 (PMC13434253; doi:10.2196/85403)
Supplement: Supplementary file 1 [file jmir_v28i1e85403_app1.docx]

# Multimedia Appendix 1: STROBE Checklist for Cross-Sectional Studies

This checklist demonstrates compliance with the Strengthening the Reporting of Observational Studies in Epidemiology (STROBE) guidelines for cross-sectional studies (von Elm et al., 2007).

| **Item** | **STROBE Recommendation** | **Location in Manuscript** |
| --- | --- | --- |
| **1** | (a) Indicate the study's design with a commonly used term in the title or the abstract | Title: “Mobile App Use and Pregnancy Health Literacy: A Cross-Sectional Study Using Bayesian Network Analysis”  Abstract (Methods): "This cross-sectional survey study included 998 pregnant and early postpartum women..." |
|  | (b) Provide in the abstract an informative and balanced summary of what was done and what was found | Abstract: Structured abstract with Background, Objective, Methods, Results, and Conclusions sections |
| **2** | Explain the scientific background and rationale for the investigation being reported | Introduction: Presents health literacy importance, digital interventions, theoretical frameworks (CLT/ICALM), and methodological approach |
| **3** | State specific objectives, including any prespecified hypotheses | Introduction (final paragraph): "This study aims to: (1) compare health literacy levels... (2) identify psychological and social-cognitive pathways... (3) validate these pathways to inform the design..." |
| **4** | Present key elements of study design early in the paper | Methods (Study design and participants): "This study employed a cross-sectional observational survey design..." |
| **5** | Describe the setting, locations, and relevant dates, including periods of recruitment, exposure, follow-up, and data collection | Methods (Procedure): "The survey was administered online... between October and December 2024" targeting "females aged 18-45 years... in the United States" |
| **6** | (a) Give the eligibility criteria, and the sources and methods of selection of participants | Methods (Study design and participants): Details stratified sampling, eligibility criteria (age 18-45, pregnant or 0-12 months postpartum), app usage qualification (>2 months, several times/week) |
| **7** | Clearly define all outcomes, exposures, predictors, potential confounders, and effect modifiers. Give diagnostic criteria, if applicable | Methods (Measures): Detailed definitions for digital engagement, emotions, health literacy, support level, support sources, health behaviors, and demographic variables. Measure provenance specified for each variable (study-specific vs. adapted from framework)" |
| **8** | For each variable of interest, give sources of data and details of methods of assessment (measurement). Describe comparability of assessment methods if there is more than one group | Methods (Measures): Each subsection describes measurement method (e.g., 7-point Likert scales for emotions, 10-point scales for health literacy, composite variable creation for digital engagement). Full questionnaire in Multimedia Appendix 2. Except for the health literacy items, all measures were purpose-designed for this survey and have not been independently validated. |
| **9** | Describe any efforts to address potential sources of bias | Methods (Procedure): "Quality control measures were implemented throughout the survey, including attention checks and open-ended validation questions to ensure data integrity." Discussion (Limitations) addresses cross-sectional design limitations and retrospective assessment concerns |
| **10** | Explain how the study size was arrived at | Methods (Study design and participants): "A total sample of 900 respondents was planned, with stratified sampling to ensure representation across three user groups... while allowing balanced stratification. " |
| **12** | (a) Describe all statistical methods, including those used to control for confounding | Methods (Statistical Analysis): Describes group comparisons performed using chi-square and Welch’s t-tests Bayesian network approach, PCA for dimensionality reduction, discretization, three-phase construction/validation process, bootstrap resampling, k-fold cross-validation. “In Bayesian networks, confounding is addressed through ... model parsimony while ensuring balanced group representation.” |
|  | (b) Describe any methods used to examine subgroups and interactions | Methods (Study design and participants): Stratification by user groups (Pregnancy+, other apps, non-users). No post-stratification weighting was applied; the quota sampling approach was designed to ensure balanced representation at the recruitment stage rather than through post-hoc statistical adjustment.  Results: Comparative analyses across groups presented |
|  | (c) Explain how missing data were addressed | Methods (Procedure): "Only responses from participants who fully completed the survey were used for analyses. Participants selecting 'prefer not to say' for health behavior variables were excluded" |
|  | (d) If applicable, describe analytical methods taking account of sampling strategy | Methods (Study design and participants): Describes stratified sampling strategy. Bayesian network approach accounts for complex relationships between variables |
|  | (e) Describe any sensitivity analyses | Methods (Network construction and validation, Phase 3): "Bootstrap resampling (n=1,000)... Sequential removal of arcs... The final optimized network was compared against the initial theory-informed structure and alternative configurations" |
| **13** | (a) Report numbers of individuals at each stage of study | Results (Participant descriptives): Reports final sample (n=998) and breakdown by groups (Pregnancy+ n=362, other apps n=507, non-users n=129) |
|  | (b) Give reasons for non-participation at each stage | Methods (Procedure): "Only responses from participants who fully completed the survey were used for analyses" |
|  | (c) Consider use of a flow diagram | Not applicable - simple cross-sectional design without complex exclusions |
| **14** | (a) Give characteristics of study participants and information on exposures and potential confounders | Results (Participants): Table 1 presents demographic characteristics, digital engagement, emotions, support, health literacy, and health behaviors by user group |
|  | (b) Indicate number of participants with missing data for each variable of interest | Methods (Procedure): Complete case analysis used. Participants with missing data excluded from analysis |
| **15** | Report numbers in each exposure category, or summary measures of exposure | Results: Table 1 reports digital engagement levels by user group. Bayesian network results presented in Figure and Table 3 |
| **16** | (a) Give unadjusted estimates and, if applicable, confounder-adjusted estimates and their precision | Results (Bayesian Network): Table 3 presents χ² and BIC scores for all arcs with p-values. Network structure inherently accounts for confounding through directed relationships |
|  | (b) Report category boundaries when continuous variables were categorized | Methods (Network construction and validation): "All continuous variables were discretized into categorical variables using distribution histograms with approximately equal group sizes" |
|  | (c) Consider translating estimates of relative risk into absolute risk for a meaningful time period | Not applicable - study examines relationships rather than risk estimation |
| **17** | Report other analyses done | Results: Reports PCA for dimensionality reduction, bootstrap resampling for arc strength evaluation, k-fold cross-validation for model performance assessment |
| **18** | Summarize key results with reference to study objectives | Discussion (Principal findings): Summarizes main finding that apps work through indirect emotional and support pathways rather than direct effects |
| **19** | Discuss limitations of the study, taking into account sources of potential bias or imprecision | Discussion (Limitations): Discusses emotional assessment limitations, Bayesian network constraints, data collection methods, cross-sectional design limitations |
| **20** | Give a cautious overall interpretation of results considering objectives, limitations, multiplicity of analyses, results from similar studies, and other relevant evidence | Discussion: Interprets findings in context of CLT and ICALM theories, discusses alignment with existing literature, acknowledges cross-sectional design prevents causal inferences |
| **21** | Discuss the generalizability (external validity) of the study results | Discussion (Implications): Discusses practical applications for app developers and healthcare providers. Limitations section acknowledges US sample and cross-sectional design. Conclusions suggest future research in diverse populations |
| **22** | Give the source of funding and the role of the funders for the present study | Conflict of Interest section: Declares 3 of the authors employed by Philips MCC who owns Pregnancy+ |
