# Supplementary material for: Mobile App Use and Pregnancy Health Literacy: Cross-Sectional Study Using Bayesian Network Analysis
Source: J Med Internet Res. 2026 Jul 20;28:e85403. doi: 10.2196/85403 (PMC13434253; doi:10.2196/85403)
Supplement: Supplementary file 2 [file jmir_v28i1e85403_app2.docx]

# Multimedia appendix 2. Survey questions

1. What is your gender?

- Male
- Female
- Other

2. Please indicate your age

_____ years old

3. Which of the following best describes your race/ethnic background?

*Please select all that apply.*

- Native American or Alaskan Native
- Asian or South Asian
- African American or Black
- Native Hawaiian or other Pacific Islander
- Caucasian or White
- Hispanic / Latino
- Middle Eastern or North African
- Other, please specify
- I’d rather not say à

4. Are you currently **pregnant**?

- Yes
- No

5. How **far along** are you in your pregnancy?

Please round up to the nearest week

- … Weeks

6. Do you have any children [excluding the one(s) you are currently pregnant with]?

- Yes
- No

7. How many children do you have?

- 1
- 2
- 3 or more

8. What is the age of your **youngest** child?

- 0-3 months
- 4-6 months
- 7-9 months
- 10-12 months
- Between 13 -18 months
- Between 19 – 24 months
- 2 years or older

**Digital engagement**

9. [Have you downloaded / Did you download] any **pregnancy apps** (e.g., BabyCenter, Ovia Pregnancy, Pregnancy+) during your pregnancy?

- Yes
- No

10. Which pregnancy app(s) did you download?

*Please select all that apply.*

- Pregnancy+
- Flo Health App
- What to Expect
- Ovia Pregnancy
- BabyCenter
- Glow Nurture
- The Bump
- Other, please specify
- None of the above

11. Which of these apps do/did you use **most often** during your pregnancy?

…….

12. In which trimester were you when you downloaded the following app(s)?

Before I got pregnant	1st trimester	2nd trimester	3rd trimester

13. How long [have you been using / did you use] [name app] [for / during your pregnancy]?

- Less than 1 month
- 1-2 months
- 3-4 months
- 5-6 months
- 7-9 months
- More than 9 months
- I have just downloaded it, and not really used it

14. How regularly, on average, [do/did] you use the [name app] app?

- Several times a day
- Several times a week
- Once a week
- 1-2 times per month
- 1-2 times every three months
- Less often
- Other, please specify:

15. How did you conceive your [youngest, if they have multiple children] child/children [the child/children you are currently pregnant with, if pregnant]?

- Naturally
- Assisted Reproduction (IVF, IUI, Fertility treatment)
- Donor or Surrogacy (Donor, Surrogacy)
- Other
- Prefer not to say

16. How long into your pregnancy was your [youngest, if they have multiple children] child born?

- Full term (37 weeks or more)
- Preterm (between 32 and 36 weeks)
- Very preterm (less than 32 weeks)

17. How confident are you in your ability to find and understand information related to your pregnancy and prenatal care?

- Not confident at all
- Not very confident
- Neutral
- Confident
- Very confident

**Emotional state**

18. Please indicate to which extent you felt each of the following emotions during the **1^st^ trimester** (< 13 weeks and 6 days) of your pregnancy.

- Stressed - Excited
- Worried – Hopeful
- Angry – Calm
- Distressed – Joyful
- Sad – At ease
- Lonely – Connected

19. Please indicate to which extent you felt each of the following emotions during the **2^nd^ trimester** (14 weeks – 27 weeks and 6 days) of your pregnancy.

- Stressed - Excited
- Worried – Hopeful
- Angry – Calm
- Distressed – Joyful
- Sad – At ease
- Lonely – Connected

20. Please indicate to which extent you felt each of the following emotions during the **3^rd^ trimester** (28 weeks – 40 weeks and 6 days) of your pregnancy.

- Stressed - Excited
- Worried – Hopeful
- Angry – Calm
- Distressed – Joyful
- Sad – At ease
- Lonely – Connected

21. Please indicate to what extent you felt each of the following emotions **following the birth of your child**.

- Stressed - Excited
- Worried – Hopeful
- Angry – Calm
- Distressed – Joyful
- Sad – At ease
- Lonely – Connected

**Prenatal visits attendance**

22. How many of your **prenatal visits** (e.g., prenatal scans, health checks, social worker checks, etc...) did you attend during your pregnancy?

*Please give an estimate in % from 0-100.*

___________ % of my prenatal visits

23. When did you, or are you planning to, have your ***first* prenatal check-up**?

- In my 1st trimester (< 13 weeks and 6 days)
- In my 2nd trimester (14 weeks – 27 weeks and 6 days)
- In my 3rd trimester (28 weeks – 40 weeks and 6 days)
- I am not planning on having it

24. What proportion of your **prenatal visits** (e.g., prenatal scans, health checks, social worker checks, etc...) that you were **supposed to attend up to this point have you actually attended**?

*Please give an estimate in % from 0-100.*

___________ % of my prenatal visits planned so far

25. What proportion of the **upcoming** **prenatal visits** (e.g., prenatal scans, health checks, social worker checks, etc...) are you intending to attend?

*Please give an estimate in % from 0-100. If you haven’t planned any (more) prenatal visits, please indicate 100%.*

___________ % of my upcoming prenatal visits

26. What was the reason(s) why you [did not (if postpartum) / did not, or are planning to not attend (if currently pregnant)] one or more of your prenatal appointments?

- Financial constraints
- Work or time conflicts
- I forgot about it
- Transportation issues
- Fear or anxiety
- It felt unnecessary
- Childcare issues
- Cultural or religious beliefs
- Miscommunication
- Previous negative experiences
- Language barriers
- Fatigue (exhaustion, sleep deprivation, etc.)
- Physical challenges (pain or difficulties to leave home)
- I changed doctor / HCP
- I rescheduled
- Other, please specify
- Prefer not to say

27. Did you or do you plan to go for the **postpartum visit** with your healthcare provider within the **first 12 weeks** of the birth of your child?

- Yes
- No
- I’m not sure

28. What reasons, if any, do you have for not going or not being sure whether you would go to your **postpartum visit** with your healthcare provider?

- Financial constraints
- Work or time conflicts
- Transportation issues
- Fear or anxiety
- It [feels / felt] unnecessary
- Childcare issues
- Cultural or religious beliefs
- Miscommunication
- Previous negative experiences
- Language barriers
- Fatigue (exhaustion, sleep deprivation, etc.)
- Physical challenges (pain or difficulties to leave home)
- I changed doctor / HCP
- No health insurance
- Other, please specify
- Prefer not to say

**Health Literacy**

29. How often [do/did] you find it **difficult to understand written information** about your pregnancy, such as pamphlets or instructions from your healthcare provider?
1. Always


5. Sometimes


10. Never
30. How confident [are/were] you in **understanding the results of your prenatal tests** and what they mean for your pregnancy?
1. Not at all confident
5. Moderately confident
10. Extremely confident
31. How confident [are/were] you in **asking your healthcare provider questions** about your pregnancy or prenatal care?
1. Not at all confident
5. Moderately confident
10. Extremely confident
32. How comfortable [are / were] you **discussing your concerns and questions with your healthcare provider** during your prenatal visits?
1. Not at all comfortable
5. Moderately comfortable
10. Extremely comfortable
33. How often [do/did] you **seek additional information about your pregnancy** from sources other than your healthcare provider, such as books, websites, or support groups?
1. Never
5. Sometimes
10. Very often
34. How confident [are/were] you in **making decisions about your prenatal care** based on the information you receive from various sources?
1. Not at all confident
5. Moderately confident
10. Extremely confident
35. Please indicate, on a scale of 1-10, how well you feel your knowledge was on each of the following pregnancy-related topics **at the start of your [current / most recent] pregnancy.**

*Please indicate on a scale where 1 is ‘I knew nothing at all’ and 10 is ‘I knew everything I needed to know’.*

1. My baby’s development
2. My body during pregnancy
3. The basic do’s and don’ts of pregnancy
4. The symptoms that are OK or not during pregnancy
5. Diet and nutrition during pregnancy
6. Prenatal care
7. How to speak to my healthcare provider

**Support sources and level**

36. Please indicate how much you agree with the following statement:

I [have felt / feel] supported during my pregnancy.

1. Strongly disagree
2. Disagree
3. Neutral
4. Agree
5. Strongly agree
37. What sources provided you with useful information or guidance during your pregnancy?

Informational support refers to advice, knowledge, and resources that helped you make informed decisions or understand different aspects of your pregnancy.

*Please select all that apply.*

1. Partner
2. Family & friends
3. Health care providers (doctors, midwives, antenatal classes etc.)
4. Pregnancy app(s)
5. Pregnancy websites / blogs
6. Social media / Influencers (e.g. Instagram, Facebook, YouTube, TikTok)
7. Documentaries, videos etc. on pregnancy
8. Pregnancy books / magazines
9. Local groups (e.g. NCT groups, childcare centers, specific gym classes)
10. Health insurance company
11. Health insurance resources (e.g., doctor visits, ultrasounds, lab tests, screenings etc.)
12. Other, please specify
13. None of these
38. You mentioned that you used health insurance resources during your pregnancy.

Which specific resources offered by health insurance providers did you use?

*Please select all that apply.*

- Doctor visits
- Ultrasounds
- Screenings (e.g., blood tests, glucose tests, genetic screenings, gestational diabetes screenings, ect.)
- Lab tests to monitor health of mother and baby
- Prescription drugs (e.g. prenatal vitamins or medications for pregnancy-related conditions)
- Therapy or counselling offers for pregnancy-related conditions (e.g., anxiety or depression)
- Telehealth consultations
- Wellness and education offers (e.g., childbirth classes, nutrition advice, support groups, etc.)
- Other, please specify
- None of these

39. Which source [do / did] you use **most often** during your pregnancy?

*Please select and rank up to your top [2/3] sources that provided you with helpful information, with 1 being the source you used most frequently.*

40. Which source [do / did] you find the **most useful** during your pregnancy?

*Please select and rank the top [2/3] sources that you find most useful, with 1 being the source most useful.*

41. What sources do you use for support (e.g. managing, support emotionally, etc…) **following** your pregnancy? *Please select all that apply.*

- Partner
- Family & friends
- Health care providers (doctors, midwives, antenatal classes etc.)
- Parenting / baby app(s) (e.g., Baby+, Baby Tracker etc.)
- Parenting / baby websites / blogs
- Social media / Influencers (e.g. Instagram, Facebook, YouTube)
- Documentaries, videos etc. on parenting / babies
- Parenting / baby books
- Parenting / baby magazines
- Local groups (e.g. NCT groups, childcare centers, specific gym classes)
- Health insurance company
- Health insurance resources (e.g., newborn health check-ups, screenings, breastfeeding counseling etc.)
- Other, please specify
- None of these

**Health behaviors**

42. Which **healthy habits** are you trying to adopt during your pregnancy, or have you already adopted prior to your pregnancy?

|  | I had already adopted this habit before my pregnancy | I have adopted this habit during my pregnancy | I’m trying / I would like to adopt this habit | I’m not trying to adopt this habit | Unsure / prefer not to say |
| --- | --- | --- | --- | --- | --- |
| **Paying attention to my** **multivitamins intake** (folic acid, iron, calcium and vitamin D) |  |  |  |  |  |
| **Moderate exercise** (at least 150 minutes each week e,g, brisk walking, swimming) |  |  |  |  |  |
| A **balanced diet** with fruits, vegetables, grains, protein and dairy |  |  |  |  |  |
| **Better stress management** (e.g., meditation, breathing exercises, and journaling) |  |  |  |  |  |
| **Reducing harmful habits** (e.g. poor diet, smoking, alcohol consumption) |  |  |  |  |  |
| **Better management of symptoms** (e.g. back ache, nausea) |  |  |  |  |  |

43. Which **healthy habits** did you adopt **following the birth of your most recent child**?

|  | I had already adopted this habit before my child was born | I successfully adopted this habit following the birth of my child | I haven't fully adopted this habit but I'm trying to | I didn’t try to adopt this habit | Unsure / prefer not to say |
| --- | --- | --- | --- | --- | --- |
| **Regular Exercise**: Try to include active moment during my day (10 to 30 min a day) as soon as I feel ready. |  |  |  |  |  |
| **Balanced Diet**: Consuming a balanced diet rich in fruits, vegetables, grains, protein, and dairy to support recovery and maintain energy levels. |  |  |  |  |  |
| **Better stress management** (e.g., meditation, breathing exercises, and journaling) |  |  |  |  |  |
| **Adequate Hydration**: Staying hydrated by drinking plenty of water throughout the day to support overall health and milk production for breastfeeding mothers. |  |  |  |  |  |
| **Adequate Sleep**: Getting enough rest, aiming for 7-9 hours of sleep per night, even if it means napping when the baby sleeps, to support both physical and mental well-being. |  |  |  |  |  |
| **Regular Check-ups:** Scheduling and attending regular postpartum check-ups with a healthcare provider to monitor physical and emotional health and address any concerns. |  |  |  |  |  |

44. Do you believe **app name** has improved how informed / educated you feel about your pregnancy?
1. No, not at all
2. …
3. …
4. …
5. Yes, a lot
45. Please indicate, on a scale of 1-10, how well you feel your knowledge on each of the following pregnancy-related topics is **now.**

- My baby’s development
- My body during pregnancy
- The basic do’s and don’ts of pregnancy
- The symptoms that are OK or not during pregnancy
- Diet and nutrition during pregnancy
- Prenatal care
- How to speak to my healthcare provider

46. Please indicate, on a scale of 1-10, to what extent do you feel [app name] **helped you to gain knowledge** on the following pregnancy-related topics**.**

- My baby’s development
- My body during pregnancy
- The basic do’s and don’ts of pregnancy
- The symptoms that are OK or not during pregnancy
- Diet and nutrition during pregnancy
- Prenatal care
- How to speak to my healthcare provider

47. Regarding your **use of [app name]** to help you during your pregnancy, to what extent do you feel the app helped you with adopting the following healthy behaviors? *Please indicate on a scale where 1 is ‘It did not help at all’ and 10 is ‘It helped enormously’.*

- Paying attention to my multivitamins intake (e.g. folic acid, iron, calcium and vitamin D)
- Moderate exercise (at least 150 minutes each week e,g, brisk walking, swimming etc…)
- A balanced diet with fruits, vegetables, grains, protein and dairy
- Better stress management (e.g., meditation, breathing exercises, and journaling)
- Reducing harmful habits (e.g. poor diet, smoking, excessive alcohol consumption, etc…)
- Better management of symptoms (e.g. back ache, nausea, etc…)

48. Regarding your use of **[app name]** to help you during your pregnancy, to what extent do you feel that the app helped you with the following aspects of your emotional wellbeing?

- The app helped me with reducing stress
- The app helped me with reducing anxiety and worry
- The app helped me with managing low mood and depressive symptoms
- The app helped me with improving my ability to control my emotions
- The app helped me with being happier
- The app helped me with having better emotional wellbeing
- The app helped me with improving my self esteem
- The app helped me with improving my confidence
- The app helped me with improving relationships with others

49. You mentioned that you use(d) [app name] during your pregnancy.
    Below is a list of features that an app like this may have. Thinking of the **features that [are/were] available to you**, which are your **most preferred** ones? *Please select all that apply.*

- 3D models / videos / illustrations of the baby’s development
- Pregnancy timeline
- Appointment calendar / reminders
- Weight gain tracker
- Baby size tracker
- Baby weight tracker
- Pregnancy symptom tracker
- Health and wellness content
- App user community access
- Other, please specify
- None of these

**App usage (only presented to women reporting pregnancy app use)**

50. Do you use the free version of **[app name]**, or are you paying a subscription for the premium version?

- Free
- Premium, and I pay for it
- Premium, covered by my health insurance
- I don’t know

51. How long have you been paying for the premium subscription to **[app name]**?

- Less than a month
- Over a month
- 2-3 months
- 4-6 months
- 7 months or more

52. How likely do you think it is that you will upgrade to the premium version of **[app name]** before the end of your pregnancy?

*Please indicate on a scale where 1 is ‘Very unlikely and 10 is ‘Very likely’.*

53. You indicated that you’re unlikely to upgrade to the premium version of [app name] before the end of your pregnancy. Which, if any, of the following are reasons why you’re unlikely to upgrade?

Please select all that apply.

- It’s too expensive
- I’m not convinced that the benefits and additional features are worth the money
- I’m already content with the free version
- I’ve read negative reviews about the premium version
- I’m not satisfied enough with the free version to want to pay extra for the premium
- I need more information on what Premium offers and the costs to know if I would be likely to upgrade
- Other, please specify:
- Don’t know [exclusive]

54. Which benefits or features, if any, do you feel would persuade you to pay for the premium subscription to [app name]? Please be as specific as possible

------------

55. Are you interested in a paid subscription to a premium version of [app name]?

- No, I am not interested
- Maybe, but I need more information on what Premium offers and how much it costs
- Maybe, but only if it’s part of my health insurance benefit/ costs covered by my health insurer
- Yes, I want to subscribe but I did not do so yet

55. Which benefits do / did you experience from using the premium version of [app name] instead of the free version? Please be as specific as possible.
56. On a scale of 1-10, how likely are you to recommend [app name] to a friend or family member?

1.	Very unlikely / 10. Very likely

**Demographics**

57. In which state of U.S. territory do you reside?
58. How many people (including yourself) live in your household?
59. Which of the following categories best describes the **total net annual income** of your household?

*Total net annual income is the income of your household following any deductions (e.g. tax)*

- Less than $39,999
- $40,000 - $79,999
- $80,000 - $119,999
- $120,000 - $159,999
- $160,000 - $199,999
- $200,000 or more
- Prefer not to say

60. What is your level of education?

- Some high school, no diploma
- High school graduate, diploma, or the equivalent (e.g., GED)
- Associate’s degree
- Bachelor’s degree
- Master’s degree
- Doctorate degree
- Other (please specify)
- I prefer not to say

61. What is your current employment status?

- Employed, full time
- Employed, part time
- Temp worker
- Self-employed
- On parental leave
- Stay-at-home
- Out of work and looking for work
- Out of work but currently not looking for work
- Retired
- Prefer not to say

62. How much time, in total, did you take off / do you intend to take off for maternity leave?

- Less than a month
- 1 month
- 2-3 months
- 4-6 months
- 7-9 months
- 10-11 months
- 12-18 months
- 19 - 24 months
- More than 2 years
- Don't know
- Not applicable

63. Which of the following provide you with your health insurance?

- My / my partner’s employer
- Via Medicaid
- Through Veteran Affairs healthcare
- I source my own insurance
- Other, please specify:
- I do not currently have health insurance
- Prefer not to say
