# Supplementary material for: Mobile App Use and Pregnancy Health Literacy: Cross-Sectional Study Using Bayesian Network Analysis
Source: J Med Internet Res. 2026 Jul 20;28:e85403. doi: 10.2196/85403 (PMC13434253; doi:10.2196/85403)
Supplement: Supplementary file 3 [file jmir_v28i1e85403_app3.docx]

# Multimedia appendix 3. Whitelist constraints for Bayesian network arc specification

| From | To | Section | Reference |
| --- | --- | --- | --- |
| Parity | Digital engagement | Psychological factors influencing pregnancy health literacy | First-time mothers face additional challenges as novice learners without existing schemas (Sweller et al., 2007) |
| Parity | Support level | Psychological factors influencing pregnancy health literacy | First-time pregnancy creates unique support needs and patterns |
| Parity | Support sources | Psychological factors influencing pregnancy health literacy | Nulliparous women may seek different information sources and support networks |
| Parity | Emotions | Psychological factors influencing pregnancy health literacy | First-time mothers experience heightened emotional responses and vulnerability to mood disturbances (Biaggi et al., 2016) |
| Digital engagement | Support level | Digital interventions for pregnancy health literacy | Apps can help overcome limited consultation time and provide supportive presence (Skar et al., 2018) |
| Digital engagement | Support sources | Digital interventions for pregnancy health literacy | Digital interventions can facilitate connections to healthcare providers, family, and peers |
| Digital engagement | Emotions | Digital interventions for pregnancy health literacy | Users continue using apps that meet emotional needs but disengage when apps fail to provide emotional support (Ding & Chai, 2015); app usage patterns predict and influence emotions (Hung et al., 2016; Mehrotra et al., 2017) |
| Digital engagement | Health literacy | Digital interventions for pregnancy health literacy | Digital interventions can improve pregnancy health literacy and enhance understanding of health topics (Hao et al., 2023; Kim et al., 2016; Karamolahi et al., 2021) |
| Support level | Support sources | Psychological factors influencing pregnancy health literacy | Feeling supported influences, the variety of support sources women access and their utilization patterns |
| Support level | Emotions | Psychological factors influencing pregnancy health literacy | Social support mediates the relationship between mental health and emotional well-being, buffering negative effects of stress (Bahrami‐Samani et al., 2023; Nguyen et al., 2022) |
| Support level | Health literacy | Psychological factors influencing pregnancy health literacy | Support from healthcare providers can improve health literacy through better utilization of health information (Nicoloro-SantaBarbara et al., 2017) |
| Support sources | Emotions | Psychological factors influencing pregnancy health literacy | Greater perceived social support from multiple sources has been associated with better emotional outcomes (Mitchell et al., 2022) |
| Support sources | Health literacy | Psychological factors influencing pregnancy health literacy | Higher levels of social support were directly linked to health literacy in women with gestational diabetes (Tang et al., 2023) |
| Emotions | Health literacy | Psychological factors influencing pregnancy health literacy | Bidirectional relationship between emotions and health literacy; pregnancy stress negatively predicts pregnancy health literacy (Zhang, Wu, et al., 2025) |
| Emotions | Nutrition | Psychological factors influencing pregnancy health literacy | Poor mental health is associated with unhealthy behaviors, while positive emotions can promote healthier nutrition choices (Auerbach et al., 2014; Hill et al., 2016) |
| Emotions | Physical activity | Psychological factors influencing pregnancy health literacy | Emotional states influence health behaviors including physical activity during pregnancy (Nguyen et al., 2022) |
| Emotions | Stress management | Psychological factors influencing pregnancy health literacy | Emotional regulation becomes intrinsic cognitive load when essential for decision-making; emotions directly influence stress management capacity (Plass & Kalyuga, 2019) |
| Health literacy | Nutrition | Pregnancy health literacy and outcomes | Health literacy shapes maternal lifestyle choices and health behaviors including dietary practices (Nawabi et al., 2021; Pratley, 2016) |
| Health literacy | Physical activity | Pregnancy health literacy and outcomes | Direct implications for health behaviors such as physical activity adherence (Gaupšienė et al., 2023) |
| Health literacy | Stress management | Pregnancy health literacy and outcomes | Enhanced health literacy results in increased empowerment and self-efficacy for health decisions, improving stress management (Tavananezhad et al., 2022; Khajeei et al., 2022) |
