# Supplementary material for: Mobile App Use and Pregnancy Health Literacy: Cross-Sectional Study Using Bayesian Network Analysis
Source: J Med Internet Res. 2026 Jul 20;28:e85403. doi: 10.2196/85403 (PMC13434253; doi:10.2196/85403)
Supplement: Supplementary file 4 [file jmir_v28i1e85403_app4.docx]

# Multimedia Appendix 4. Histograms with discretization of emotions, support sources, digital engagement and health literacy

Number of participants

Digital engagement

Health literacy

Support sources

Emotions

2

4

6

-1

0

1

20

40

60

80

0

20

40

60

80

0

30

60

90

0

50

100

0

0

10

20

2

4

6

8

10
